# Determinants of compliance with anti-vectorial protective measures among non-immune travellers during missions to tropical Africa

**DOI:** 10.1186/1475-2875-10-232

**Published:** 2011-08-10

**Authors:** Emmanuel Sagui, Noémie Resseguier, Vanessa Machault, Lénaïck Ollivier, Eve Orlandi-Pradines, Gaetan Texier, Frédéric Pages, Remy Michel, Bruno Pradines, Sébastien Briolant, Alain Buguet, Catherine Tourette-Turgis, Christophe Rogier

**Affiliations:** 1 Field Infectiology Department, Institute for Biomedical Research of the French Armed Forces (IRBA) & URMITE UMR6236, Allée du Médecin Colonel Jamot, Parc du Pharo, BP60109, 13262 Marseille Cedex 07, France; 2 Hôpital d'Instruction des Armées Laveran, BP 60149, 13384 Marseille Cedex 13, France; 3 Département d'épidémiologie et de santé publique & EA3283, Parc du Pharo, 13262 Marseille Cedex 07, France; 4 EA4170 Free Radicals, Energy Substrates and Physiopathology, Claude-Bernard Lyon I University, 8 Avenue Rockefeller, 69373 Lyon Cedex 08, France; 5 Institut d'éducation thérapeutique, Fondation partenariale, University Pierre et Marie Curie, Paris 6, and University of Rouen, France; 6 Institut Pasteur de Madagascar, B.P. 1274, 101 Antananarivo, Madagascar

## Abstract

**Background:**

The effectiveness of anti-vectorial malaria protective measures in travellers and expatriates is hampered by incorrect compliance. The objective of the present study was to identify the determinants of compliance with anti-vectorial protective measures (AVPMs) in this population that is particularly at risk because of their lack of immunity.

**Methods:**

Compliance with wearing long clothing, sleeping under insecticide-impregnated bed nets (IIBNs) and using insect repellent was estimated and analysed by questionnaires administered to 2,205 French military travellers from 20 groups before and after short-term missions (approximately four months) in six tropical African countries (Senegal, Ivory Coast, Chad, Central African Republic, Gabon and Djibouti). For each AVPM, the association of "correct compliance" with individual and collective variables was investigated using random-effect mixed logistic regression models to take into account the clustered design of the study.

**Results:**

The correct compliance rates were 48.6%, 50.6% and 18.5% for wearing long clothing, sleeping under bed nets and using repellents, respectively. Depending on the AVPM, correct compliance was significantly associated with the following factors: country, older than 24 years of age, management responsibilities, the perception of a personal malaria risk greater than that of other travellers, the occurrence of life events, early bedtime (*i.e*., before midnight), the type of stay (field operation compared to training), the absence of medical history of malaria, the absence of previous travel in malaria-endemic areas and the absence of tobacco consumption.

There was no competition between compliance with the different AVPMs or between compliance with any AVPM and malaria chemoprophylaxis.

**Conclusion:**

Interventions aimed at improving compliance with AVPMs should target young people without management responsibilities who are scheduled for non-operational activities in countries with high risk of clinical malaria. Weak associations between compliance and history of clinical malaria or variables that pertain to threat perception suggest that cognition-based interventions referencing a "bad experience" with clinical malaria could have only a slight impact on the improvement of compliance. Further studies should focus on the cognitive and behavioural predictors of compliance with AVPMs.

## Background

Each year, approximately 25 to 30 million travellers from temperate countries visit malaria-endemic areas, and about 30,000 of them suffer from travel-associated clinical malaria [1]. Travellers can be protected from malaria by using chemoprophylaxis and anti-vectorial protective measures (AVPMs), including using insect repellents, wearing long clothing at night with long sleeves and pants that provide full coverage, and sleeping under insecticide-impregnated bed nets (IIBNs) [2].

Because *Plasmodium falciparum *and *Plasmodium vivax *may be resistant to most drugs used in chemoprophylaxis, AVPMs should be considered as the first line prevention against malaria among non-immune travellers. Regardless of the preventive measures employed, effectiveness depends on their appropriate usage, including user's compliance [3-8]. The determinants of incorrect compliance with chemoprophylaxis have been associated with several individual factors [7,9-13]. In contrast, few studies have focused on incorrect compliance with AVPMs [5,7,11]. Poor compliance with AVPMs has been associated with age greater than 50 years [5] and a length of stay in endemic countries longer than one month [11].

Non-immune travellers sent to malaria-endemic areas for professional purposes, such as building sites, opencast working, tree-felling, plantations, humanitarian operations within governmental or non-governmental organizations (NGOs), or military operations, can contract malaria, and their risk increases if they work in field conditions [14-17]. To improve compliance with AVPMs, workers must commit to complying with prophylactic measures. Whatever the working conditions, a better understanding of anti-vectorial prophylactic behaviours could improve AVPM implementation and become the basis for interventions that target factors related to lack of compliance.

The aim of the present study was to identify the determinants of compliance with AVPMs among short-term (*i.e*., 4-5 months) professional travellers in African malaria-endemic areas.

## Methods

### Study design and inclusion criteria

This prospective cohort study was carried out between February 2004 and September 2007 among 20 French military groups. Each group was composed of 72 to 189 individuals and was selected to represent various conditions that these travellers could experience in Africa. All members of these groups served on a short-term mission (approximately four months) in one of the following African countries: the Ivory Coast (n = 8 groups), Gabon (n = 4 groups), Chad (n = 3 groups), the Central African Republic (n = 2 groups), Senegal (n = 2 groups) and Djibouti (n = 1 group). Individuals were required to take malaria chemoprophylaxis and apply AVPMs.

All members of these groups were eligible for the study and were invited to participate. Two self-administered questionnaires were filled out by each individual and validated during a face-to-face interview with a member of the research team. The first survey was administered no earlier than 15 days before their departure, the second being completed no later than 15 days after returning home. People who were absent on the dates of the surveys or who left their group between the first survey and their departure for Africa or during their stay in the field were excluded from the analysis.

### Data collection

Dependent variables included the level of compliance with the following AVPMs: using IIBNs while sleeping, using insect repellents and wearing long clothing at night (*i.e*., when individuals were outside the IIBN). For each of the above three AVPMs, the level of compliance was assessed as either "never", "seldom", "often" or "always". People spent their night time in one of the following locations and activities: in the base without professional activity (*i.e*., resting), in the base with professional activity (*i.e*., night guard), outside the base with professional activity (*i.e*., field operation) and outside the base without professional activity (*i.e*., on free time). For each of the three dependant variables, a global score was calculated by weighting the compliance with AVPMs in each situation by the time spent in each of these locations and activities.

For long clothing and IIBN use, "correct" compliance (as a binary variable) was defined as a global score reflecting responses such as "always", while "incorrect" compliance was defined as one of the three other possible responses: "often", "seldom" or "never". In the case of insect repellent, the prevalence of a global score suggesting an "always" response was unexpectedly low, which could have hampered calculations. The "correct" compliance for this AVPM was therefore defined as a global score reflecting an "always" or "often" response. For each individual, independent variables that could be potential determinants of compliance were recorded upon either departure or return.

Mission-specific variables included the dates of departure and return, country and type of stay, either for field operations (Ivory Coast, Central African Republic and Chad) or training, which occurred mainly in urban conditions (Senegal, Djibouti and Gabon). Demographic variables included age, gender, management responsibilities and previous travel overseas to other malaria-endemic areas. Behavioural variables included tobacco consumption, wake and sleep time during the stay and usual morningness or eveningness determined by the Horne and Ösberg Morningness-Eveningness Questionnaire [18,19]. Morningness and eveningness were used to determine chronotype. Chronotypes were split into two groups. Morning individuals are those who wake up early and are more alert during the first part of the day. Evening individuals are those who tend to go to bed later and are more alert during the latter part of the day. Social or familial variables included the following events that occurred during the stay of subjects in Africa: birth of a child, illness or death of a relative or friend, and separation or divorce.

Cognitive variables concerned subjective items including perceived risk of malaria during the stay, perceived frequency of mosquito bites and perceived individual attractiveness for mosquitoes. The perceived risk of malaria during the stay was associated with three conditions: perceived individual likelihood of acquiring clinical malaria, severity of the disease if malaria were personally contracted and a dangerous situation of any possible nature.

Variables relative to medical history or medical events during the African stay included presence of clinical malaria, clinical dengue fever, allergy to insect bites and usual cutaneous response to insect bites. Cases of clinical malaria were recorded by the military weekly disease epidemiological surveillance system and defined as clinical attacks occurring during the stay or within 16 weeks after returning to France with biological confirmation of plasmodial infection (*i.e*., positive thin blood smear, positive quantitative buffy coat malaria diagnosis system test, or histine rich protein 2 rapid diagnostic test).

The variable pertaining to chemoprophylaxis was the declared compliance. It was defined as "correct" if no dose was reported missed for the entire stay on the return questionnaire. Otherwise, it was considered "incorrect" (*i.e*., any of three other possible responses: "less than one missed dose per month", "one or more missed doses per month, but less than one missed dose per week" or "one or more missed doses per week").

### Statistical methods

Data were recorded using EpiDATA, version 3.0 (http://www.EpiData.dk) and checked for consistency before analysis using SAS version 9.1.3 (SAS Institute Inc., Cary, NC, USA) and STATA 9.0 (StataCorpLP, College Station, TX, USA). Subjects were considered in the statistical analysis only if they completed both questionnaires. Missing values affected less than 1% of the participants and were replaced using a single imputation method. When a question was not asked to one or more groups, the responses were coded as missing data for those groups.

The confidence intervals for "correct" compliances were estimated by taking into account the clustered design of the study using the "svy" commands under STATA 9.0. Compliance with AVPM was analysed as a dependent variable according to individual and group characteristics using a random-effect mixed logistic regression model with the GLIMMIX procedure under SAS. The model was designed to take into account the intra-group correlations that could exist due to the sampling design (*i.e*., group effect as random effect). The logistic model was also adjusted using a generalized estimating equations (GEE) approach. Random-effect and GEE regression models allowed for the estimation of group-specific and population-averaged effects, respectively [20].

First, a descriptive analysis of the dependant and independent variables was performed. A bivariate analysis was then conducted by entering each independent variable into the random-effect mixed logistic regression models. Variables were retained for the multivariate analysis when their effect had a p-value below 0.25 [21]. A backward stepwise selection procedure was applied to retain significant (p < 0.05) independent variables and their interactions in the final model. The statistical quality of the final model was assessed by looking at the difference between observed and predicted probabilities for "correct" compliance.

### Ethical considerations

The protocol was approved by the Marseille II Ethics Committee (advice no. 02/81, 12/13/2002). Informed consent was obtained from each participant at the beginning of the study after a thorough explanation of the study's purpose.

## Results

### Study population

Of the 3,051 eligible individuals from the 20 groups surveyed, 846 were excluded because they did not complete both questionnaires, leaving a working sample of 2,205 subjects. The most common reason for not completing the questionnaires was the absence of the subject at the time of the survey (due to holidays, training, mission or transfer to another group or service), which was independent of the purpose of the study and the dependent variables. Some individuals who had planned to go to Africa and completed the first questionnaire did not travel and were replaced by others who had not completed the first survey. Some individuals returned to France before the rest of their group or were transferred to another city and were not present at the time of the second survey.

The descriptive characteristics of the groups are summarized in Table 1. A total of 24 clinical malaria cases occurred among the 2,205 individuals during and after the stay in malaria-endemic areas, which corresponds to an incidence rate of 1.09 cases per 100 subject-missions (2.98 cases per 100 subject-years in malaria-endemic areas).

**Table 1:** Characteristics of the groups and malaria cases

Group	Country of stay	Dates of stay (MM/YY) Start End		Type of stay	Nb of subjects	Nb of men	Age: median (25%-75% quantile)	Nb of clinical malaria cases during or after the stay
1	Ivory Coast	02/04	06/04	Field	144	143	26 (25 - 29)	3
2	Djibouti	01/05	05/05	Training	115	114	23 (27 - 30)	0
3	CAR	02/05	05/05	Field	115	115	26 (23 - 29)	2
4	Ivory Coast	02/05	05/05	Field	108	108	22 (21 - 26)	1
5	Ivory Coast	02/05	05/05	Field	95	95	22 (20 - 24)	1
6	Ivory Coast	02/05	05/05	Field	73	72	23 (22 - 26)	1
7	Senegal	06/05	09/05	Training	133	132	26 (22 - 30)	0
8	Ivory Coast	06/08	09/05	Field	84	84	25 (22 - 28.5)	0
9	Ivory Coast	06/08	09/05	Field	72	66	24 (22.5 - 28.5)	1
10	Chad	10/05	01/06	Field	134	130	24 (21 - 27)	2
11	Senegal	02/06	05/06	Training	102	100	25 (22 - 28)	0
12	Chad	02/06	05/06	Field	82	77	25 (22 - 29)	1
13	Gabon	07/06	11/06	Training	93	93	23.5 (21- 29)	1
14	Gabon	07/06	11/06	Training	113	113	24 (21 - 29)	2
15	Chad	06/06	09/06	Field	189	185	24 (21 - 29)	0
16	Gabon	12/06	04/07	Training	125	123	25 (22 - 30)	0
17	Gabon	12/06	04/07	Training	135	133	24 (22 - 31)	1
18	CAR	06/07	09/07	Field	102	102	23 (20 - 29)	1
19	Ivory Coast	06/07	09/07	Field	94	92	22 (20 - 25)	3
20	Ivory Coast	06/07	09/07	Field	97	96	23 (21 - 28)	4
Total		02/04	09/07		2205	2173	24 (22 - 29)	24

Nb: number; CAR: Central African Republic.

The "correct" compliance rates were 48.6% (95% confidence interval (95% CI): 46.5 - 50.7%; ranging from 2.6% to 88.2%), 50.6% (95% CI: 48.5 - 52.7%; ranging from 1.7% to 97.3%) and 18.5% (95% CI: 16.8 - 20.1%; ranging from 4.9% to 59.6%) for wearing long clothing at night, using IIBN while sleeping and using insect repellents, respectively. Gender, rainy season, mosquito bite burden, perceived mosquito attractiveness compared with other people and perception of the severity of malaria were not associated with compliance with any of the AVPMs in bivariate analyses.

#### Compliance with long clothing at night

Of the responses corresponding to incorrect compliance, the compliance with wearing long clothing at night was scored as "often" in 497 individuals (22.5%), "seldom" in 407 individuals (18.5%) and "never" in 230 individuals (10.4%).

In the final mixed multivariate logistic regression model, "correct" compliance was significantly associated with the country of the stay (higher in Ivory Coast and Central African Republic than in the other countries), age over 24, management responsibilities, occurrence of any familial event, medical history of malaria and "correct" compliance with the use of IIBN and chemoprophylaxis (Table 2). "Correct" compliance with wearing long clothing at night was not significantly associated with "correct" compliance with the use of insect repellent (adjusted odds ratio (OR) 1.29; 95% CI 0.96 - 1.73; p = 0.0927). Replacing the country by the type of stay did not significantly change the final model; individuals involved in field operations were more likely to be compliant with wearing long clothing than those who were not involved in field operations (adjusted OR 7.13; 95%, CI: 2.51 - 20.2; p < 0.001).

**Table 2:** Multivariate logistic regression analysis for wearing long clothing at night

	Nb	CC	Unadjusted OR			Adjusted OR		
			
			OR	95% CI	p value	OR	95% CI	p value
Country					< .0001			< .0001
Ivory Coast (field)	767	600	1			1		
Djibouti (training)	115	3	0.007	0.001 - 0.04		0.01	0.00 - 0.08	
Gabon (training)	466	143	0.12	0.05 - 0.26		0.15	0.07 - 0.32	
CAR (field)	217	174	1.09	0.38 - 3.13		1.14	0.43 - 3.05	
Senegal (training)	235	33	0.05	0.02 - 0.13		0.06	0.02 - 0.16	
Chad (field)	405	118	0.08	0.03 - 0.19		0.11	0.05 - 0.27	
Age								0.0138
18 - 24 y.	1113	498	1		< .0001	1		
25 y. and more	1092	573	1.77	1.40 - 2.22		1.40	1.08 - 1.80	
Management responsabilities					< .0001			0.0006
No	1703	774	1			1		
Yes	502	297	2.11	1.61 - 2.76		1.79	1.33 - 2.41	
Occurrence of any familial event during the stay					0.0229			0.0276
No	1934	931	1			1		
Yes	271	140	1.49	1.06 - 2.09		1.48	1.05 - 2.10	
Previous medical history of malaria					0.0545			0.0315
No	1964	951	1			1		
Yes	196	95	1.60	1.09 - 2.33		1.57	1.05 - 2.36	
Use of IIBN while sleeping during the stay					< .0001			0.0008
Not always	1090	322	1			1		
Always	1115	749	2.02	1.51 - 2.70		1.78	1.32 - 2.41	
Chemoprophylaxis during the stay					0.0002			0.0084
No md or < 1/month	1634	932	1			1		
< 1 md/week	322	107	0.69	0.49 - 0.96		0.79	0.56 - 1.11	
≥ 1 md/week	249	32	0.34	0.21 - 0.56		0.45	0.27 - 0.74	
Random effect (ie group effect)								< 0.05

Nb: Number of subjects; CC: Number of subjects with correct compliance; CAR: Central African Republic;

CI: confidence interval; IIBN: insecticide impregnated bed net; md: missed dose.

#### Compliance with the use of IIBN

Of the responses corresponding to "incorrect" compliance, the compliance with the use of IIBNs was scored as "often" in 227 individuals (10.3%), "seldom" in 385 individuals (17.6%) and "never" in 478 individuals (21.7%).

In the final mixed multivariate logistic regression model, "correct" compliance with the use of IIBNs was significantly associated with the country of the stay (higher in Ivory Coast and lower in Djibouti, Gabon and Chad), absence of tobacco consumption, bedtime before midnight and "correct" compliance with both wearing long clothing at night and chemoprophylaxis (Table 3). "Correct" compliance with the use of IIBNs was not significantly associated with "correct" compliance with the use of insect repellent (adjusted OR 1.22; 95% CI 0.86 - 1.73; p = 0.2602).

**Table 3:** Multivariate logistic regression analysis for the use of insecticide-impregnated bed net while sleeping

	Nb	CC	Unadjusted OR			Adjusted OR		
			
			OR	95% CI	p value	OR	95% CI	p value
Country					< .0001			0.0003
Ivory Coast (field)	767	643	1			1		
Djibouti (training)	115	2	0.003	< 0.001 - 0.04		0.006	< 0.001 - 0.11	
Gabon (training)	466	176	0.08	0.02 - 0.34		0.14	0.03 - 0.65	
CAR (field)	217	164	0.47	0.08 - 2.80		0.44	0.06 - 3.08	
Senegal (training)	235	120	0.16	0.03 - 0.93		0.37	0.05 - 2.64	
Chad (field)	405	10	0.003	< 0.001 - 0.02		0.003	< 0.001 - 0.02	
Tobacco consumption					0.0060			0.0216
Non smoker	995	488	1			1		
Smoker	1210	583	0.69	0.53 - 0.89		0.73	0.56 - 0.95	
Bedtime during the stay					0.0049			0.029
Before or at midnight	1698	843	1			1		
After midnight	363	143	0.54	0.38 - 0.77		0.61	0.43 - 0.87	
NA (group 1)	144	129	8.21	0.04 - > 999.9		1.18	0.09 - 15.54	
Wearing long clothing at night during the stay					0.0001			0.001
Not always	1134	366	1			1		
Always	1071	749	1.96	1.46 - 2.63		1.74	1.29 - 2.35	
Chemoprophylaxis during the stay					< .0001			< .0001
No md or < 1/month	1634	907	1			1		
< 1 md/week	322	137	0.49	0.34 - 0.71		0.54	0.37 - 0.78	
≥ 1 md/week	249	71	0.29	0.19 - 0.45		0.33	0.21 - 0.52	
Random effect (*i.e*. group effect)								< 0.02

Nb: Number of subjects; CC: Number of subjects with correct compliance; CAR: Central African Republic;

CI confidence interval; NA: not available; md: missed dose.

#### Compliance with the use of repellents

Of the responses corresponding to "incorrect" compliance, the compliance with the use of insect repellents was scored as "seldom" and "never" in 695 individuals (31.5%) and 1103 individuals (50%), respectively. "Correct" compliance was scored as "always" and "often" in 328 individuals (14.9%) and 79 individuals (5.6%), respectively.

In the final mixed multivariate logistic regression model, "correct" compliance with use of insect repellents was significantly associated with age over 24, absence of previous overseas travel in malaria-endemic areas, perception of personal malaria risk greater than that of other individuals and "correct" compliance with wearing long clothing at night (Table 4). It was not significantly associated with either "correct" compliance with the use of IIBNs (adjusted OR 1.33; 95% CI 0.96 - 1.85; p = 0.085) or "correct" compliance with the use of chemoprophylaxis (< 1 missed dose/week: adjusted OR 0.72, 95% CI 0.48 - 1.07; ≥ 1 missed dose/week: adjusted OR 0.58, 95% CI 0.35 - 0.97, p = 0.063).

**Table 4:** Multivariate logistic regression analysis for the use of insect repellents

	Nb	CC	Unadjusted OR			Adjusted OR		
			
			OR	95% CI	p value	OR	95% CI	p value
Age					0.0524			0.0155
18 - 24 y.	1113	199	1			1		
25 y. and more	1092	208	1.28	0.99 - 1.63		1.42	1.08 - 1.87	
Previous overseas travel in malaria endemic areas					0.1696			0.0192
No	541	122	1			1		
Yes	1664	285	0.83	0.63 - 1.09		0.69	0.50 - 0.93	
Perception of personal malaria risk					0.0095			0.0088
Inferior/others	333	48	0.79	0.55 - 1.14		0.78	0.54 - 1.12	
Equal/others	1441	250	1			1		
Superior/others	431	109	1.44	1.08 - 1.91		1.44	1.08 - 1.91	
Wearing long clothing at night during the stay					0.0146			0.0143
Not always	1134	173	1			1		
Always	1071	234	1.44	1.08 - 1.91		1.44	1.09 - 1.92	
Random effect (ie group effect)								< 0.005

Nb: Number of subjects; CC:Number of subjects with correct compliance;

CI confidence interval.

With each AVPM, the estimates of the regression coefficients for the variables retained in the final models were not significantly changed after the withdrawal of compliance with chemoprophylaxis, the use of the two other AVPMs, or the use of a GEE approach. The group effect was significant in the three final models (p < 0.05).

## Discussion

In endemic areas, malaria is a major health burden for employees of international companies, including expatriates and other non-immune workers. During the construction of the 1,000 km-pipeline from Chad oilfields to the coast of Cameroon, up to 18.5% of workers suffered from malaria 12 months after the start of construction [14]. Malaria was also the main health issue among employees of an oil palm plantation [16]. Other examples of expatriates from non-endemic areas working in malaria-endemic areas are members of the Red Cross or other humanitarian NGOs, employees of private companies, including stopover crews, employees of governmental and international organizations and military personnel, all of whom represent non-immune travellers.

Resistance of *P. falciparum *to most anti-malarial drugs makes the appropriate use of AVPMs even more important. Few studies have assessed compliance with AVPMs. Compliance has mostly been studied as a secondary outcome in the context of assessment of compliance with chemoprophylaxis [4,5,7,9,11,22]. In the present study, which was conducted in a large sample of professional travellers, compliance with wearing long clothing at night, using IIBNs and using insect repellents was the primary outcome.

The prevalences of "correct" compliance with wearing long clothing and the use of IIBNs were more than 2.5 times higher than the prevalence of "correct" compliance with the use of insect repellents. The need for repeated applications of repellents could explain this difference from other AVPMs. Moreover, the social and staff pressures to be compliant with visible measures (*i.e*., wearing long clothing and using IIBNs) may be higher than the pressure to comply with non-visible measures, such as the use of repellents.

### Absence of competing compliance

Some studies have shown no association [13] or even competition [9] between the use of malaria prophylactic measures. In the present study, there was no competing compliance between the three AVPMs. "Correct" compliance with one out of the three AVPMs was associated with "correct" compliance with one or two of the other AVPMs in bivariate or in multivariate analyses. Moreover, there was a significant association between "correct" compliance with any AVPM and the reported "correct" compliance with chemoprophylaxis with a dose-effect relationship (*i.e*., having good compliance with chemoprophylaxis increased the odds of having "correct" compliance with AVPMs). These findings are in agreement with previous reports [12,17,23] that suggest common pathways for prophylactic behaviours.

### Individual factors

In a French cohort of 899 travellers [5], age over 50 years was associated with poor AVPM compliance. In the present study, age over 24 years, being a staff member and the occurrence of a life event during the stay were associated with "correct" compliance with one or several AVPMs. These individual factors associated with "correct" compliance could be explained by increased maturity, which could lead to better awareness of the danger of malaria.

Bedtime after midnight was associated with "incorrect" compliance with the use of IIBNs and long clothing. In other studies, eveningness, a concept closely related to bedtime after midnight, has been associated with "incorrect" compliance with chemoprophylaxis [12]. Eveningness is a multi-factorial variable with genetic and environmental components [24] and has been associated with certain personality traits and dimensions, such as extraversion, novelty seeking, impulsivity and anti-conformism [25]. These results favour the hypothesis that there are common determinants of incorrect compliance, of which late bedtime could be a marker.

The perception of personal malaria risk and severity comprises the concept of threat perception, one of the two aspects of health behaviours in the "Health Belief Model" [26]. In a previous study, the "Health Belief Model" combined with measures of perceived behavioural control was able to explain nearly half of the variance in reported adherence among mefloquine users and 40% of the variance among chloroquine and proguanil users [3]. Moreover, travellers who perceived themselves to be at higher malaria risk were likely to be more compliant with malaria chemoprophylaxis [3,11-13]. In the present study, the perception of lower personal malaria risk compared with other individuals was associated with "incorrect" compliance with the use of insect repellent but not with the other AVPMs, and the perception of the severity of malaria was not associated with compliance with any of the three AVPMs. Therefore, the perception of personal malaria risk and severity (*i.e*., the two aspects of individual representation of health behaviours in the "Health Belief Model") did not appear to have the same relationship with compliance with chemoprophylaxis. In accordance with the "Health Belief Model", these results suggest that even if the perceived probability of a threat and its perceived severity are the first two dimensions generating perception of risk and motivation for action, they are insufficient to predict any health behaviour. Perceived barriers appear to be the most powerful predictor of health action [27].

Medical history of malaria was associated with "correct" compliance with wearing long clothing and with "incorrect" compliance with the other AVPMs and chemoprophylaxis [12]. These findings are comparable with the behaviours of patients with chronic diseases [28,29]. Past experience of disease burden is insufficient to adopt appropriate behaviours against relapses. The weak associations between compliance with AVPMs and both a medical history of malaria and the perception of personal malaria risk and severity suggest that interventions based on cognitive approaches in reference to "bad experiences" could have little impact on compliance improvement.

### Collective factors

Participating in field operations was associated with "correct" compliance with the three AVPMs. The same finding applied for chemoprophylaxis [12], suggesting that the two have the same determinants. In field operation-type stays, individuals may better perceive the risks and need for protection and may be under higher pressure from the staff to comply with security rules to prevent casualties in the context of a dangerous environment.

The prevalence of "correct" compliance with wearing long clothing and using IIBNs was higher in countries where the incidence rate of clinical malaria was known to be higher (*i.e*., from 7.5 and 9.0 clinical malaria cases/100 person-years in the Ivory Coast and Central African Republic, respectively) than in the other countries (from 0.1 to 2.1 clinical malaria cases/100 person-years in Djibouti and Gabon, respectively) during the 2005-2006 period, according to the French Forces epidemiological surveillance. Individually driven compliance and staff pressure to ensure "correct" compliance could be modulated according to the environment and the perceived or documented level of malaria risk. More favourable living conditions, such as sleeping in permanent structures or air-conditioned rooms during training or less intrusive missions, could have made individuals feel safer towards mosquito bites, thus impairing their compliance with AVPMs. Because a country was the theatre of only one type of stay (either for field operations or training), it was not statistically feasible to segregate the two variables.

The group effect remained significant in all three final multivariate models. Furthermore, the rate of compliance was heterogeneous between groups. These results suggest that individual behaviours were probably not independent within a group and that significant collective behaviour determinants were not identified in the present study.

Although this study was performed among French military personnel travelling for a four-month period in tropical Africa, the present results could be directly extrapolated to other aforementioned groups of non-immune workers staying in malaria-endemic areas. Extrapolation to individual travellers staying for a shorter duration is less straightforward due to the supposed absence of collective factors. However, data about compliance with AVPMs are scarce among such populations [5,7,11]. Thus, the present study could elucidate some of the determinants of the compliance of travellers with AVPMs. Moreover, a similar study to be conducted among civilian travellers would be difficult to perform, mainly because of sample size (more than 2,000 people in the present study), heterogeneity in data collection and exposure to malaria risk. Nevertheless, further studies should focus on cognitive and behavioural predictors of compliance of travellers with AVPMs.

## Competing interests

The authors declare that they have no competing interests.

## Authors' contributions

ES participated in the data analysis and drafted the manuscript. NR, VM and LO participated in the data management and in the data analysis. EOP, GT, FP, RM, BP and SB participated in the data collection. LO, EOP, GT, FP, RM, AB, CTT and CR participated in the design of the study. CR conceived the study and coordinated the data collection, the data management, the data analysis and the redaction of the manuscript. All authors read and approved the final manuscript.
